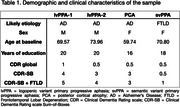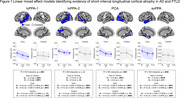# Detecting short‐interval longitudinal cortical atrophy in neurodegenerative dementias via cluster scanning: A proof of concept

**DOI:** 10.1002/alz.094630

**Published:** 2025-01-09

**Authors:** Yuta Katsumi, Michael Brickhouse, Lindsay C. Hanford, Jared A. Nielsen, Maxwell L. Elliott, Ross W. Mair, Alexandra Touroutoglou, Mark C. Eldaief, Randy L. Buckner, Bradford C. Dickerson

**Affiliations:** ^1^ Massachusetts General Hospital and Harvard Medical School, Boston, MA USA; ^2^ Harvard University, Cambridge, MA USA; ^3^ Brigham Young University, Provo, UT USA

## Abstract

**Background:**

Regional brain atrophy estimated from structural MRI is the most widely used measure of neurodegeneration in Alzheimer’s disease (AD), Frontotemporal Lobar Degeneration (FTLD), and other dementias. Yet, traditional MRI‐derived morphometric estimates are susceptible to measurement errors, posing a challenge for reliably detecting longitudinal atrophy, particularly over short intervals. We examined the utility of multiple accelerated MRI scans acquired in rapid succession (i.e., “cluster scanning”) for detecting longitudinal cortical atrophy over 3‐ and 6‐month intervals within individual patients.

**Method:**

Four individuals with mild cognitive impairment or mild dementia likely due to AD or FTLD participated (see Table 1 for demographic and clinical characteristics). At baseline, 3 months, and 6 months, structural MRI data were collected on a 3 Tesla scanner using a fast multi‐echo (ME) MPRAGE sequence (acquisition time = 2’23’’, TR = 2200ms, TEs = 1.57/3.39/5.21/7.03ms, field of view = 192×192mm^2^, 144 slices, 4x GRAPPA acceleration, 1.2mm isotropic voxels). At each timepoint, participants underwent 32 MEMPRAGE scans acquired in four sessions over two days. Each MRI acquisition was processed with FreeSurfer version 6.0.0 using the recon‐all pipeline to derive a sample of estimates of cortical thickness at each timepoint. An independent, standard MEMPRAGE scan acquired prior to this study was used to define vertex‐wise regions of interest (ROI) masks for each participant compared to a group of cognitively unimpaired participants, representing phenotypically vulnerable regions expected to show longitudinal atrophy (“core” atrophy ROIs) and regions with no atrophy (“control” ROIs).

**Result:**

Figures 1 summarizes the results obtained from a linear mixed effects model constructed separately for each participant. We found a Timepoint x ROI interaction in three of the four participants (all *p≤*0.0049), driven primarily by core regions exhibiting longitudinal atrophy of a greater magnitude from baseline to 3 months (n = 3) and to 6 months (n = 2) compared with control regions. In all four participants, core regions showed longitudinal atrophy from baseline to 3 months and to 6 months (all *p*<0.0001).

**Conclusion:**

These findings provide proof‐of‐concept evidence that pooling multiple morphometric estimates derived from fast MRI acquired in clusters enables the detection of cortical atrophy over short intervals in individual patients with neurodegenerative dementias.